# Mitochondria: A Potential Rejuvenation Tool against Aging

**DOI:** 10.14336/AD.2023.0712

**Published:** 2024-04-01

**Authors:** Qian Hua Phua, Shi Yan Ng, Boon-Seng Soh

**Affiliations:** ^1^Institute of Molecular and Cell Biology (IMCB), Agency for Science, Technology and Research (A*STAR), Proteos, Singapore.; ^2^Department of Biological Sciences, National University of Singapore, Singapore.; ^3^National University of Singapore, Yong Loo Lin School of Medicine (Department of Physiology), Singapore.; ^4^National Neuroscience Institute, Singapore.

**Keywords:** aging, mitochondria, mitotherapy, lifespan, healthspan

## Abstract

Aging is a complex physiological process encompassing both physical and cognitive decline over time. This intricate process is governed by a multitude of hallmarks and pathways, which collectively contribute to the emergence of numerous age-related diseases. In response to the remarkable increase in human life expectancy, there has been a substantial rise in research focusing on the development of anti-aging therapies and pharmacological interventions. Mitochondrial dysfunction, a critical factor in the aging process, significantly impacts overall cellular health. In this extensive review, we will explore the contemporary landscape of anti-aging strategies, placing particular emphasis on the promising potential of mitotherapy as a ground-breaking approach to counteract the aging process. Moreover, we will investigate the successful application of mitochondrial transplantation in both animal models and clinical trials, emphasizing its translational potential. Finally, we will discuss the inherent challenges and future possibilities of mitotherapy within the realm of aging research and intervention.

## Introduction

Aging is a complex phenomenon that involves multi-factorial degeneration at a cellular, organismal and molecular level. Biological aging correlates with an overall physiological decline of functional tissues and organs, which eventually results in a myriad of age-related pathologies. These include Alzheimer’s disease (AD), Parkinson’s disease (PD), cardiovascular diseases (CVD), cancer, hearing loss, and osteoarthritis, amongst many others [1]. At a cellular and molecular level, aging has been associated with increased oxidative stress, accumulations of DNA modifications, epigenetic alterations, and organelle damage [2, 3]. Amongst the array of hallmarks associated with aging, there has been a growing interest in the role of mitochondria and how its defects can influence aging phenotypes.

The mitochondrion is a multi-faceted organelle that contributes to diverse functions including cellular metabolism, intracellular signaling, and immune response [4]. With advanced age, there is an associated increase in mitochondria perturbations due to the gradual build-up of mitochondrial DNA (mtDNA) mutations, increased reactive oxygen species (ROS), impaired respiratory chain activities, and altered mitochondrial dynamics [5]. Disruption in mitochondrial dynamic affects mitophagy, hence contributing to the accumulation of defective mitochondria [6]. Altogether, given the tight interconnections among the numerous pathways, will accelerate the decline of tissue function and ultimately hasten the aging process.

## The interplay between mitochondria and the aging process

The mitochondria free radical theory of aging proposes that as individual ages, there is a buildup of ROS that results from oxidative phosphorylation that drives further mitochondria deterioration and cellular damage, which accelerates the aging process [3, 7]. The overproduction of ROS causes oxidative damage to the mtDNA, mitochondrial proteins, respiratory complexes, and mitophagy [8, 9]. Supporting this, a study observed a significant increase in the activity of manganese-dependent superoxide dismutase (MnSOD) in human skeletal muscles with advancing age [10]. Another study by Ji et al. further suggested that heightened MnSOD activity serves as a potential defense mechanism against elevated ROS levels [11]. Over time, the interplay between aging, heightened ROS levels, and impaired mitochondrial function creates a vicious cycle that accelerates the aging process, thus affecting lifespan and healthspan.

However, it is also important to note that ROS also plays a crucial role in activating signalling pathways that are involved in proliferation and survival, in response to age-induced stress. For instance, studies have shown that activation of stress-resistance pathways by ROS can enhance the lifespan of organisms such as *C. elegans* and *S. cerevisiae* [12, 13]. Considering these findings, it is plausible that age-associated declines are observed only when ROS levels surpass a certain threshold. This indicates that ROS may have a dual role, acting as both damaging agents and important mediators of stress response pathways.

Mitochondria DNA mutations, accumulate with age and are particularly evident in energetically demanding organs including the brain, skeletal muscle, retina, ovaries, hepatocytes, and heart [14]. While these mutations are randomly distributed across the mitochondrial genome, aged tissues exhibit a higher occurrence of base-substitution, whereas post-mitotic tissues tend to show a greater prevalence of large-scale deletions [15]. Over time, a multitude of studies have collectively shown the presence of different mitochondrial deletions in a broad range of age-related diseases. Among them, the most extensively studied modification is the 4977-bp deletion in mtDNA (mtDNA4977) [16]. This deletion has been observed to accumulate as individuals age and is detected in various tissues of older individuals, with a higher frequency in tissues characterized by high oxygen consumption [17, 18] Similar to humans, a 3867-bp deletion analogous to the 4977-bp deletion was also observed to accumulate in aged mice [19].

In the context of neurodegeneration, patients with PD were found to express higher levels of mtDNA deletions in their striatum and substantia niagra as compared to normal aging individuals [20, 21]. Similarly, mtDNA deletions were found in AD mice models, resulting in disruptions in mitochondria function [22]. Besides neurodegenerative diseases, ragged red fibers (RRF) within skeletal muscles becomes increasingly prominent with advanced age. These RRF serve as a compensatory response to mitochondrial respiratory capacity impairments. Notably, in the later stages of aging, a substantial portion of these RRF fibers exhibit a significant mtDNA deletion, which correlates with a deficiency in cytochrome c oxidase activity, an important component of the mitochondrial respiratory chain. These findings strongly indicate that mtDNA deletions play a pivotal role in the progression of aging-related sarcopenia [23]. In the context of aging-related heart failure, mutated mice models which encompass high levels of mtDNA deletions exhibit a range of cardiac aging phenotypes. These include cardiac hypertrophy and dilatation, mosaic pattern due to cytochrome *c* oxidase deficiency, perturbations in diastolic and systolic function, as well as cardiac fibrosis [24-26].

Around 1% of the mitochondrial proteome, including 13 vital proteins in respiratory chain complexes, is encoded by mtDNA [14]. Throughout an individual’s lifetime, mtDNA mutations build up, resulting in complex defects, impaired oxidative phosphorylation, and cellular dysfunction [27]. The first evidence demonstrating the direct impact of mtDNA mutations on premature aging came from the creation of "mtDNA-mutator mice," which express a proof-reading-deficient version of the catalytic subunit of polymerase gamma (PolgA) [24]. These mutator mice contain a high level of mtDNA point mutations and deletions, which is thought to be closely associated with the expression of pre-mature aging phenotypes such as age-related hearing, hair and muscle loss [24, 26]. Interestingly, in the mutator mice's liver and heart mitochondria, complexes I, III, and IV were significantly downregulated, possibly due to accumulated mtDNA point mutations causing amino acid substitutions in mtDNA-encoded respiratory subunits, impairing protein structure. Consequently, these alterations disrupt complex assembly, leading to respiratory chain deficiency and promoting premature aging phenotypes [28].

In relation to this, heteroplasmy, which refers to the co-expression of mutant and wild type mtDNA copies within a cell, serves as an indicator for measuring the level of mtDNA mutations. When heteroplasmy exceeds a certain threshold, tissues become energetically compromised, resulting in an undesirable loss of function. A population study conducted on individuals over 70 years of age discovered an increase in a particular mitochondrial mutation, m3243A>G [29]. Elevated heteroplasmy at this site was found to be associated with an increased risk of dementia-related and stroke mortality [29]. A separate study conducted by the same group also reported that increased heteroplasmy level at 20 different disease-causing mtDNA sites was correlated with decreased cognitive function, hearing, vision and mobility function in elderly individuals [30]. These studies suggest that mtDNA heteroplasmy could serve as a valuable biomarker for age-related function.

Mitochondria are highly complex and dynamic organelles that are constantly undergoing fusion and fission events. The process is vital to facilitate essential cellular functions such as adaptation to nutrients availability, mitigate mitochondrial trafficking which aids in Adenosine 5’-triphosphate (ATP) production, and most importantly the maintenance of a well-interconnected mitochondrial network [3, 31]. Earlier studies conducted on animal models such as flies and nematodes reported the presence of enlarged and fragmented mitochondria, effects of disrupted dynamics [32, 33]. In Drosophila, inclination toward fission was reported during aging. Increased fission activity causes fragmented mitochondria, which disrupt cellular homeostasis and signaling. This, in turn, results in germline stem cell loss and ultimately leads to tissue degeneration. [34]. Considering the significance of preserving mitochondrial architecture integrity in aging, attaining a well-balanced fusion and fission process is essential for increased longevity.

Mitophagy, a subset of autophagy, in which mitochondria are selectively degraded, is a highly vital process that regulates the quality of mitochondria through the elimination of poorly or non-functional mitochondria. The maintenance of mitochondria quality is essential as it affects multiple vital cellular functions such as metabolism, homeostasis and disease prevention [35]. Impaired mitophagy leads to pro-aging metabolic perturbations such as an increase in ROS and mtDNA mutation, as well as impaired ATP production [36]. Interestingly, studies reported the close association of mitophagy-related genes in longevity, as observed in model organisms. For instance, PTEN Induced Kinase 1 (PINK-1) overexpression with α-synuclein in dopaminergic neurons enhance lifespan in Drosophilia, whereas the loss of Parkin shorten lifespan [37, 38]. Similarly, in *C. elegans*, the loss of PINK1 and Parkin reduces the lifespan of mitochondria respiration mutants [39]. Apart from studying animal models, Fang et al. have observed a significant reduction in basal levels of mitophagy in the hippocampus of patients with AD. This decrease in mitophagy contributes to the accumulation of malfunctioning mitochondria and subsequently impairs cellular metabolism. In their study, Fang et al. also found that restoring mitophagy abolished tau hyper-phosphorylation in human neuronal cells associated with AD. Additionally, significant improvements were observed in cognitive decline and amyloid-beta (Aβ) pathology in both mice and *C. elegans* models of AD when mitophagy was reinstated [40]. Based on current studies and understanding, it appears that mitophagy is an essential cellular process that helps to promote lifespan.

Currently, the available evidence on mitophagy in humans is limited. To fully understand the implications of mitophagy in human cellular processes, further investigations are crucial. These additional studies will provide a deeper understanding of the significance and potential advantages associated with mitophagy in diverse human cellular processes. As researchers continue to uncover the role of mitophagy in aging, enhancing this process has emerged as a potential therapeutic target for promoting healthy aging and mitigating the effects of age-related diseases. Further studies are necessary to confirm the potential benefits and establish the most effective strategies to harness mitophagy for human health and longevity.

## Mitotherapy

### Mechanisms of mitochondrial transfer

Mitochondrial transfer is a natural phenomenon that takes place under both normal and pathogenic conditions. In 1969, Ruby et al. uncovered the concept of mitochondrial transfer when they observed the presence of mitochondria within intercellular tunnels connecting adjacent developing mouse oocytes [41]. Among the various methods of mitochondrial trafficking, tunneling nanotubes (TNTs) represent a significant route, enabling the transfer of mitochondria between distinct cell lines through a coculture system [42, 43]. TNTs consist of actin or microtubule-based cytoplasmic extensions, creating intracellular transport networks that facilitate cargo transfer [44]. When TNTs were abolished, a considerable decrease in mitochondrial transfer within cocultures was observed [45]. To support long-range intercellular communication, extracellular vesicles act as carriers, encapsulating mitochondria and releasing them into blood vessels or tissues [46, 47]. Hayakawa et al. reported that the release of extracellular mitochondrial particles by astrocytes conferred neuroprotective effects and promote neuronal viability post-ischaemic stroke [48]. Lastly, mitochondria could also be transferred via cell fusion or gap junctions formed via connexins and cytoskeleton proteins [49]. In a study conducted by Acquistapace et al., live cell imaging revealed the presence of functional mitochondria in mouse cardiomyocytes directly co-cultured with human adipose-derived stem cells [50]. The successful transfer of mitochondria through cell fusion is essential for the reprogramming of post-mitotic murine cardiomyocytes into proliferating cardiac progenitors [50]. The topic on mitochondrial mechanism has been extensively reviewed by Liu et al. [51]. Understanding how mitochondria are transferred is important as it sets the foundation for mitotherapy translational application in clinical trials.

### Current interventions targeting mitochondrial function enhancement in the context of disease and aging

**Figure 1.**
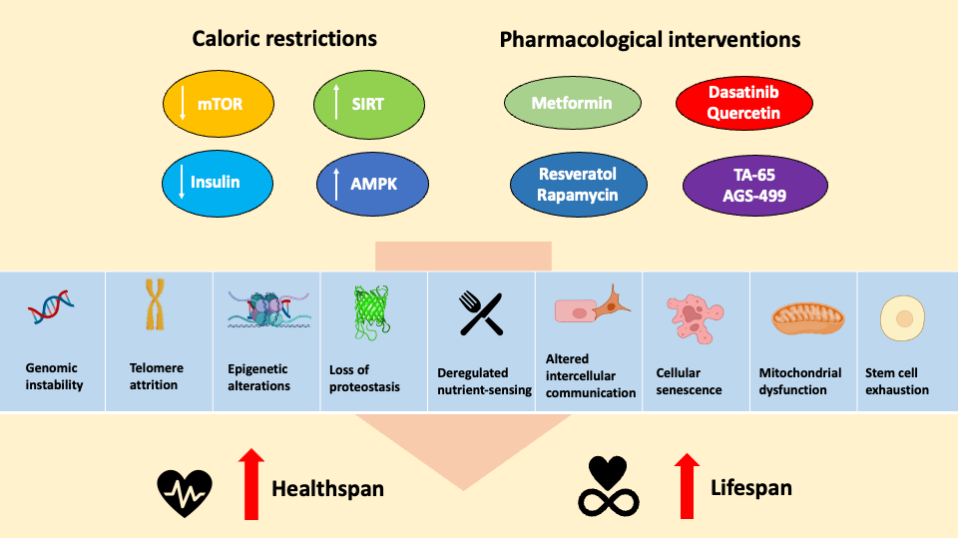
**Nutritional and pharmacological interventions against the hallmarks of aging**. This figure was created with BioRender.com.

Given the pivotal roles of mitochondria, several studies have reported the use of various approaches that could enhance mitochondria function. These approaches encompass pharmacological supplements as well as dietary interventions and exercise regimens. Caloric restriction (CR) is a widely recognized anti-aging approach that has been shown to markedly increase lifespan and delay age-related pathologies across various organisms, including non-human primates [52, 53]. Long-term caloric restriction has also been associated with a reduced incidence of chronic illnesses, including cancer, cardiovascular disease, and hypertension [54]. The effects of CR are complex and mitochondrial function is one of the key mechanisms by which CR extends lifespan (Fig. 1). Studies have shown that caloric restriction can modulate mitochondrial activity through several processes, including mitophagy, mitochondrial biogenesis, metabolic shifts as well as mitochondrial respiration [55, 56].

Nisoli et al. documented a substantial increase in mitochondrial content in the brain, heart, liver, and adipose tissue, following a 30% caloric restriction. Furthermore, the amount of mtDNA and expression of various important players of mitochondrial biogenesis such as peroxisome proliferator-activated receptor-γ coactivator 1α (PGC-1α), nuclear respiratory factor-1 (NRF-1), and mitochondrial transcription factor A (TFAM) were significantly elevated in mice fed with CR diet. These findings collectively indicate a promotion of mitochondrial biogenesis due to CR. In conjunction with the up-regulation of these genes, an increase in ATP concentration was observed, serving as an indication of enhanced respiratory activity [57].

To better understand the association between CR and metabolism, extensive research has been conducted to explore the underlying mechanisms by which CR influences the metabolic network, resulting in an increased lifespan through the stimulation of mitochondrial biogenesis. However, the precise mechanisms involved are still not fully understood. Nevertheless, SIRT1 and AMP-activated protein kinase (AMPK) have emerged as crucial factors in mediating the longevity effects associated with CR [58, 59]. Supporting this, a number of studies suggest that SIRT1 activates PGC-1α through deacetylation, ultimately enhancing mitochondrial activity [60, 61]. Furthermore, Bergeron et al. reported long-term activation of AMPK was shown to lead to the activation of NRF-1 and, consequently, an enhancement in muscle mitochondrial density [62].

In addition, CR has been shown to modulate respiratory capacity in different tissues. This includes enhancing mitochondrial respiration rate in brown adipose tissue and reducing maximal respiratory rate while limiting hydrogen peroxide release. These findings highlight the multifaceted effects of CR on cellular respiration [57, 63, 64]. In the brain, CR was revealed to effectively reduce the age-related membrane rigidization and limited the production of oxy-radicals when mitochondria were stimulated with succinate, highlighting its potential in preserving mitochondrial function in the aging brain [65]. In another study, older mice subjected to caloric restriction (CR) exhibited heightened respiration rates, indicating that CR enhances the inherent function of the respiratory chain. Interestingly, skeletal muscles from these mice undergoing caloric restriction (CR) showed lower levels of 8-oxo-dG, indicating reduced oxidative DNA damage. This implies that caloric restriction (CR) has the potential to reduce oxidative stress by improving the capacity of internal antioxidant defenses in skeletal muscles, ultimately leading to enhanced mitochondrial integrity and optimal functional performance [66].

In addition to dietary interventions, numerous studies have provided evidence that regular exercise can significantly improve mitochondrial function by increasing the content of mitochondria, enhancing oxidative phosphorylation, and improving respiratory capacity [67-71]. Endurance training has been shown to elevate the levels of mitochondrial proteins involved in β-oxidation, the tricarboxylic acid (TCA) cycle, and the electron transport chain, thereby enhancing energy capacity [68]. Remarkably, long-term exercise has been found to increase mitochondrial volume by a substantial 40-50%, accompanied by improvements in oxidative capacity and a reduction in ROS [72]. Furthermore, several studies have reported that exercise training facilitates the elimination of abnormal mitochondria, promoting the turnover of healthy mitochondria [73, 74]. Taken together, these findings demonstrate how the incorporation of CR and consistent exercise into lifestyle practices can significantly enhance mitochondrial health and cellular bioenergetics.

Other means to enhance mitochondrial function includes nutritional supplements. This includes various options such as vitamins, antioxidants, enzyme inhibitors, and co-factors [75]. Enhancing mitochondrial biogenesis to address respiratory capacity deficiencies holds promise as a potential approach for treating mitochondrial diseases. One strategy with great potential involves targeting peroxisome proliferator-activated receptors (PPARs), which play a crucial role in governing mitochondrial bioenergetics and metabolic homeostasis [4]. For instance, pioglitazone, a FDA-approved drug belonging to the thiazolidinediones (TZDs), activates PPARs, have exhibited promising outcomes in promoting mitochondrial biogenesis in diabetic patients, thereby enhancing oxidative phosphorylation (OXPHOS) activity [76, 77]. Apart from targeting PPARs, other potential targets for restoring mitochondria function involve targeting the modulation of PGC-1-α through SIRT1 and AMPK [4]. For instance, dietary supplementation of NAD+ precursors, nicotinamide mononucleotide (NMN) increased NAD+, activating SIRT1 signalling. This elevation in SIRT1 activity leads to improved mitochondrial biogenesis, enhanced mitochondrial function and a boost in both metabolic fitness and exercise endurance [78, 79]. Altogether, the exploration of pharmacological supplements targeting mitochondrial function through enhancing mitochondrial biogenesis and improving oxidative phosphorylation demonstrates promising avenues for therapeutic interventions in mitochondrial diseases.

In relation to longevity, certain compounds that mimic the effects of caloric restriction (CR), such as metformin and resveratrol, as well as rapamycin, an established mTOR signaling antagonist, have demonstrated the ability to extend both lifespan and healthspan in *C. elegans* and mouse models. These compounds target AMPK and complex 1 of the mitochondrial respiratory chain [80-82]. The effects of these interventions have been observed in mouse models that were fed a high-calorie diet and treated with resveratrol. Administration of resveratrol led to increased AMPK and PGC1-α activity, accompanied by a rise in mitochondrial abundance. Additionally, there was improvement in muscle function and enhanced insulin sensitivity, which are beneficial changes associated with longevity[83].

These findings suggest that caloric restriction and its pharmacological mimetics hold promise in positively influencing mitochondrial activity and biogenesis, which may ultimately contribute to an extended lifespan and improved health outcomes [80-82]. While CR and pharmacological interventions offer promising anti-aging benefits, CR demands considerable lifestyle changes, and pharmacological treatments may have side effects like metabolic imbalances, respiratory depression, and increased cancer risk with long-term use [84, 85]. These concerns must be thoroughly assessed for safety before implementing such interventions.

### Mitochondrial transplantation

In recent years, mitochondrial transplantation has proven to exhibit vast therapeutic benefits in disease treatment particularly metabolic, neurological and cardiac pathology [86]. The translation of bench to bedside stemmed from McCully *et al.* in 2009, where they isolated fresh mitochondria from healthy cardiac tissue and injected them into the ischemic region of the rabbits’ hearts during early reperfusion. Following the transplant, there was a notable reduction in myocardial necrosis and a significant improvement in post-ischemic function [87]. Following McCully *et al*, other studies also displayed the benefits of mitochondrial transplantation through cardiac ischemia models. In 2016, Cowan et al. displayed the ability of exogenous mitochondria to decrease myocardial infarct size and increase post-ischemia cardiac functionality through coronary vascular perfusion in a rabbit model [88]. Following, Guariento et al. demonstrated an interesting and novel concept of preischemic mitochondria transplantation which has proven to be a therapeutic strategy for prophylactic myocardial protection in porcine models of regional ischemic reperfusion injury (IRI). Through both single and serial delivery of autologous mitochondria, myocardial infarct size has been significantly reduced and cardiac function improved tremendously. Notably, the therapeutic outcomes of serial delivery and single delivery were similar in comparison [89]. Besides cardiac improvement, the therapeutic potential of mitochondrial transplantation has been demonstrated in other organs. For example, exogenous mitochondria have been demonstrated to rescue hepatocyte function in high-fat diet-induced fatty liver mice, a model of non-alcoholic fatty liver disease [90]. Similarly, enhanced lung mechanics and improved tissue recovery were observed in ischemia-reperfusion injury mice models that received mitochondrial transplantation [91]. In the most recent study by Hayashida et al., performing mitochondrial transplantation immediately following resuscitation from cardiac arrest substantially improved both survival rates and neurological recovery for rats experiencing post-cardiac arrest [92]. Existing mitochondrial transplantation in different animal models is summarised in table 1.

**Table 1:** Summary of existing mitochondria transplantation in animal models.

Targeted organs	Disease models	Methods	Types of transplant	Therapeutic outcomes	Ref.
**Brain**	Parkinson’s disease in healthy C57BL/6J mice models	Intravenous injection	Xenogeneic	• Increased ETC activity • Reduction in ROS activity • Prevention of cell apoptosis and necrosis	[120]
**Brain**	Alzheimer's disease in AD mice models	Intravenous injection	Xenogeneic	• Enhanced cognitive performance • Significant decrease in neuronal loss • Reduced gliosis in the hippocampus	[121]
**Brain**	Cerebral ischemic injury in Sprague- dawley rat models	Intracerebroventricular injection	Autologous	• Reduction in cellular oxidative stress and apoptosis • Enhanced neurogenesis • Decreased brain infarct volume • Reversed neurological deficits	[122]
**Brain**	Sprague-dawley rats with spinal cord injury	Injection into the mediolateral gray matter	Allogenic	• Maintenance of bioenergetics	[123]
**Heart**	Ischemia-reperfusion heart in New Zealand white rabbits	Injection into the ischemic zone of the heart	Allogenic	• Significant reduction in apoptosis and necrosis • Enhanced cellular viability • Myocardial functional recovery	[87]
**Heart**	Ischemia-reperfusion heart in New Zealand white rabbits	Injection into the ischemic zone of the heart	Autologous	• Reduction in myocardial necrosis and inflammatory markers • Enhanced oxygen consumption rate and high energy synthesis • Enhanced ATP	[112]
**Heart**	Ischemia-reperfusion heart in Yorkshire pigs	Injection into the left coronary ostium	Autologous	• Enhanced regional and global myocardial function • Decreased myocardial infarct size	[124]
**Liver**	Fatty liver C57BL/6J mice models	Intravenous injection	Xenogeneic	• Decreased lipid content • Restored cellular redox content • Reduction in oxidative stress	[90]
**Liver**	Ischemia-reperfusion injury in the liver of Wistar rat models	Injection into spleen	Allogenic	• Reduction in oxidative stress • Reduction in mitochondrial damage and subsequent cell death	[125]
**Lung**	Ischemia-reperfusion injury in the lung of C57BL/6J mice models	Injection into pulmonary artery	Allogenic	• Improved lung mechanics and enhances tissue recovery	[91]

In addition to animal models, mitochondrial transplantation has proven its success in human clinical studies as well. The first clinical application occurred in 2017, where healthy autologous mitochondria were harvested and isolated from nonischemic skeletal muscle and injected into the damaged myocardium of 5 pediatric patients who required central extracorporeal membrane oxygenation (ECMO) support for ischemia-reperfusion-associated myocardial dysfunction [93]. Upon the surgical procedure, none of the patients suffered from arrhythmia, 4 out of 5 patients demonstrated improvement in ventricular functions and were successfully removed from ECMO support [93]. Thereafter in 2020, the same group reported the success of autologous mitochondria transplantation in a study conducted on a greater pool of pediatric patients. Similarly, no patients suffered from adverse short-term complications such as arrhythmia or scarring [94]. Patients who received the mitochondria transplantation were successful in detaching from ECMO support and exhibited improvement in the ventricular strain [94]. Currently, there are only two clinical trials, notably from the same group of researchers, McCully et al., targeting pediatric patients suffering from ischemia-reperfusion-associated myocardial dysfunction. Further studies have to be conducted for optimal surgical process of mitochondria transplantation across different organs to better demonstrate the robustness of mitotherapy.

## Revolutionizing mitotherapy’s future with bioengineered mitochondria

To advance mitotherapy and maximize the potential of mitochondria as an innovative tool for disease treatment, multiple supplementary measures can be pursued to enhance its effectiveness in combating diverse pathologies. One promising approach involves the exploration of gene editing technology specifically targeted at correcting mtDNA genes, subsequently reducing mutant mtDNA, improved mitochondria function and ultimately eliminating mitochondrial diseases. Current tools for mitochondrial gene editing includes restriction endonucleases (RE) technology, zinc finger nuclease (ZFN) technology, transcription activator like effector nuclease (TALEN) technology and even CRISPR/Cas9 system [95]. In independent studies employing distinct mitochondrial gene editing technologies, RE and ZFN, the correction of m.8993T>G mutation has been successfully corrected. This correction resulted in a decrease in mutant mtDNA levels, restoration of ATP and wild type mtDNA levels, and the reestablishment of normal mitochondrial membrane potential (MMP) [96, 97]. In addition, another gene-editing tool, TALEN has also effectively eliminated m.3243A>G mutation in mitochondrial disease patient-specific induced pluripotent stem cells, which subsequently displayed restoration of mitochondrial respiration and bioenergetics [98]. Collectively, these gene editing tools alter the level of mtDNA heteroplasmy, eliminating the mutant fraction and ultimately resulting in enhanced mitochondrial bioenergetics. In the realm of genetic modification *in vivo*, it is also crucial to prioritize the careful design of delivery systems to prevent any unintended off-target effects. Despite the utilization of the CRISPR-Cas9 gene-editing tool, its implementation remains controversial due to numerous unresolved questions associated with off-target effects [99]. Methods to circumvent some of these off-target effects *in vivo* have been extensively discussed by Han et al. [100]. The potential to genetically correct mtDNA *in vivo* presents tremendous opportunities in enhancing the patient's cellular metabolism, leading to improved health outcomes and unparalleled efficiency in cellular energy utilization.

An alternative strategy to enhance mitotherapy involves implementing surface modifications to mitochondria, aiming to improve the uptake and specific targeting of exogenous mitochondria by desired tissue or cell types. A recent study by Nakano et al., showed that mitochondria coated with cationic and fusogenic lipids, DOTAP and DOPE exhibited elevated MMP, improved uptake and neuroprotection in neurons [101]. In addition, peptide-labelled mitochondria also enhanced delivery into dopaminergic neurons and improved locomotive activity in the PD rats [102]. Lastly, polymer functionalization of mitochondria using Dextran-triphenylphosphonium (TPP) has shown a three-fold increase in cellular internalization by cardiac cells compared to uncoated mitochondria. This uptake was accompanied by a metabolic shift from glycolytic to oxidative state and an increase in the oxygen consumption rate [103]. Although the current body of research on mitochondria as a targeted drug delivery vehicle is limited, significant advances have been achieved in the field of exosomes. In the case of cerebral ischemia therapy, cyclo(Arg-Gly-Asp-DTyr-Lys) peptide [c(RGDyK)] conjugated exosomes which contained curcumin were found to accumulate in the region of ischemic brain lesions upon intravenous injection [104]. As a result, there was a significant suppression of inflammatory response and cellular apoptosis in the lesion area. The cumulative evidence from these examples highlights the immense potential of bioengineered surface modification on mitochondria, which could open up new avenues for targeted delivery and improved cellular uptake, making it a promising and novel strategy for efficient drug delivery in the treatment of diseases, particularly those related to metabolic impairments. Collectively, the convergence of these approaches pave the way for more effective and precise interventions in treating mitochondrial-related diseases.

## Mitotherapy and aging

During aging, dysfunctional mitochondria compilation causes the deterioration of cellular and physiological health, resulting in age-related phenotypes. Hence, the replacement of aged mitochondria with healthy, mutation-free ones, presents an alternative avenue to investigate as a potential anti-aging treatment. Extrapolating from the technology of mitochondria transfer, Zhao et al. experimented with the concept of harvesting young, healthy mitochondria and injecting them into aging models. In this study, they performed intravenous injections of healthy, allogenic mitochondria isolated from young mice into aged mice and observed enhanced metabolic alterations [105]. Upon mitotherapy treatment, the level of ROS dropped, and ATP content increased significantly as compared to the aged mice. Besides metabolic improvement, there was also a functional enhancement in the cognitive and motor performance in learning and memory abilities [105]. Lastly, mitotherapy also improved mouse sports endurance, evident from longer swimming time in treated mice [105]. This study is the first to uncover new insights on mitotherapy and its huge potential in anti-aging context.

In an independent recent study by Javani et al., mitochondria obtained from young rat brain were transplanted into aged rats which resulted in the attenuation of aging-related stress induced anxiety and depressive-like behavior [106]. Studies have demonstrated a strong correlation between aging, chronic stress, and impaired mitochondria and bioenergetics. This association ultimately leads to a decrease in energy levels, which in turn contributes to the development of depression [107]. However, by introducing young mitochondria through transplantation, the levels of ATP and MMP were restored. This restoration significantly improved cellular bioenergetics and consequently led to an amelioration of age-related neurobehavioral changes [106]. While the current number of examples illustrating mitotherapy's translational application in addressing aging-related disorders is limited, with extensive research, mitotherapy holds great potential in managing and mitigating the effects of aging.

## Limitations & future perspectives

As described above, there are multiple studies which demonstrated the success of mitochondrial transplantation. However, there are also some challenges faced in the process. Firstly, isolation and storage are major concerns for the therapy to succeed. Whilst freshly isolated mitochondria are often used in mitotherapy, the storage condition plays a huge role in the organelle’s functionality. It has been reported that prolonged storage of mitochondria correlates with a decline in mitochondria respiratory capacity. Additionally, the preservation of the integrity of the membrane potential is important for mitochondria to function optimally. Even though several researchers have experimented on different storage buffers such as University of Wisconsin solution, Eurocollins solution and 4-(2-HydroxyEthyl)-1PiperazineEthaneSulfonic acid (HEPES)-sucrose-based buffer to preserve mitochondria, these are of limited effectiveness against mitochondria impairment [108, 109]. Improving mitochondria quality control is critical, therefore it would be best for a standardized protocol to be established for optimal harvest and storage conditions, whilst following the strict guidelines of safety and ethics.

Additionally, immunological response plays a vital role in the effectiveness of the therapy. Similar to other forms of surgical procedures, it is important to investigate the mechanism of immune reaction to mitochondria transplant. Significant inflammatory response was observed in a mouse heterotopic heart transplantation model, which led to eventual graft rejection [110]. When endothelial cells were exposed to extrinsic mitochondria, there was an up-regulation of adhesion molecules, and this promotes the secretion of inflammatory cytokines [110]. However, there were also studies that reported the absence of inflammatory markers elevation post-transplantation of autologous mitochondria [111-113]. While previous studies were mostly conducted using autologous mitochondria, Ramirez-Barbieri et al showed that immune response was not triggered despite the administration of syngeneic and allogenic mitochondria [113]. In addition, Acquistapace et al. also demonstrated the success of mitochondria transfer in a cross-species co-culture system between mouse and human [50]. The significance of these findings is extremely important, as they expand the potential sources of donor mitochondria for therapeutic purposes. This approach also creates a readily available supply of healthy mitochondria, thus offering a promising avenue for therapeutic translation.

**Figure 2.**
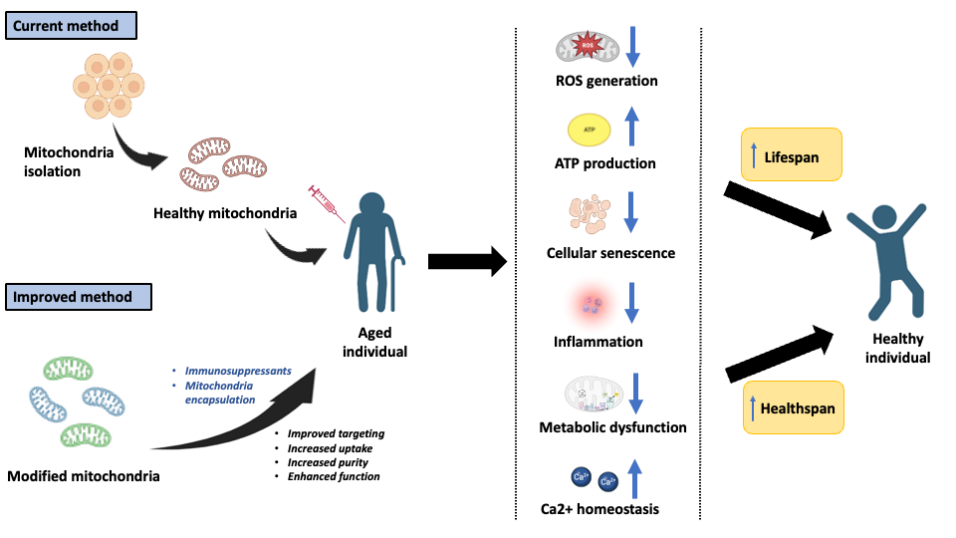
**An overview of mitotherapy’s current and future therapeutic translation**. This figure was created with BioRender.com.

To reduce the risk of an immune reaction, immunosuppressants are often co-administered with mitochondria during transfer, as has been done in previous studies. Another method to potentially reduce immune rejection could be through the encapsulation of mitochondria by microvesicles or liposomes. It has been proven that the membrane of extracellular vesicles aids in maintaining the functional integrity and lifespan of mitochondria in the bloodstream [114, 115]. To further improve the uptake and targeting efficacy, extra modifications could be made to the membranes of these transport vehicles, as discussed previously (Fig. 2).

## Ethical and safety considerations of mitotherapy

From an ethical perspective, mitotherapy generally aligns with established standards, as numerous studies have shown that personal traits are primarily determined by genes derived from nuclear DNA [116]. However, there are specific concerns related to the origin of donor mitochondria. In autologous transplantation, where mitochondria from tissue with a low risk of mtDNA mutation are used to treat the same individual, ethical concerns are rarely raised. In cases of mitochondrial allogenic transfer, preferential consideration is often given to close family members, and if not feasible, haplotype matching should be considered [117, 118]. Lastly, while xenogenic transfer has shown feasibility, it is still in its infancy stages and requires further investigation before its ethical implications can be fully understood. In-depth exploration of the ethical issues surrounding mitochondrial transplantation can be found in other review articles [116, 119].

## Conclusion

Mitochondria play a crucial role in numerous cellular processes, including metabolism, intracellular signaling, apoptosis, and immune response, making them a vital target for therapeutic interventions. The decline in mitochondrial function is closely linked to aging, as disruptions in their activity can lead to a cascade of impaired cellular pathways and quality control mechanisms, ultimately resulting in the accumulation of defective mitochondria and accelerated aging. To counteract this aging process, the transfer of healthy, functional mitochondria may serve as a potential treatment option. However, current clinical trials exploring mitotherapy for aging are scarce, highlighting the need for further research to unlock the full potential of mitochondrial therapies in aging-related applications. Addressing ethical and safety concerns will also be crucial moving forward. In summary, mitotherapy holds significant promise as an innovative approach to combat aging, paving the way for ground-breaking advancements in the improvement of healthspan and lifespan in the future.
